# Pharmacological and nonpharmacological measures of pain management and treatment among neonates

**DOI:** 10.5935/0103-507X.20190007

**Published:** 2019

**Authors:** Hanna Isa Almeida Maciel, Marcela Foureaux Costa, Anna Caroline Leite Costa, Juliana de Oliveira Marcatto, Bruna Figueiredo Manzo, Mariana Bueno

**Affiliations:** 1 Escola de Enfermagem, Universidade Federal de Minas Gerais - Belo Horizonte (MG), Brasil.; 2 The Hospital for Sick Children - Toronto, ON, Canadá.

**Keywords:** Infant, newborn, Pain/prevention & control, Drug therapy, Analgesia, Neonatal nursing, Pain management, Intensive care units, neonatal

## Abstract

**Objective:**

This study sought to describe and quantify the pharmacological and nonpharmacological strategies used to relieve the pain/stress of neonates during hospitalization in neonatal intensive care units.

**Methods:**

This quantitative, longitudinal, and descriptive study examined 50 neonates from neonatal intensive care unit admission to discharge.

**Results:**

A total of 9,948 painful/stressful procedures were recorded (mean = 11.25 ± 6.3) per day per neonate. A total of 11,722 pain-management and relief interventions were performed, of which 11,495 (98.1%) were nonpharmacological strategies, and 227 (1.9%) were pharmacological interventions. On average, each neonate received 235 pain-management and treatment interventions during hospitalization, 13 nonpharmacological interventions per day, and one pharmacological intervention every 2 days.

**Conclusion:**

Neonates receive few specific measures for pain relief given the high number of painful and stressful procedures performed during hospitalization. Thus, it is essential to implement effective pain-relief protocols.

## INTRODUCTION

Pain management among neonates presents a challenge to clinical practice. Although neonates are able to process nociceptive stimuli, painful procedures are commonly performed in neonatal care units without adequate treatment.^(^^1^^)^ Repeated and untreated pain experiences during hospitalization at such early stages of life might lead to neurodevelopmental and behavioral damage, with detrimental consequences over both the short and long term.^(^^2,3^^)^

Avoiding painful interventions should be the best strategy to manage neonatal pain. However, numerous diagnostic and therapeutic procedures are needed in neonatal intensive care units (ICUs) because they promote the stability and clinical recovery of the neonate; thus, this environment is hostile to the neonate and his or her family. In addition, frequent handling and excessive light and noise increase the amplitude of the initial painful stimulus, which can negatively affect the clinical outcome. Thus, neonates must be spared from interventions whose benefits do not outweigh the harmful effect inherent to the procedure.^(^^4-6^^)^

The multidisciplinary team, especially the nursing team, is responsible for the use of neonatal pain-relief strategies. Thus, assessing, preventing, and managing pain are important actions and should be considered during care through the adoption of pharmacological and nonpharmacological strategies.^(^^7^^)^ Despite evidence of the deleterious effects of pain among neonates and the efficacy of pharmacological and nonpharmacological pain-relief strategies, recently published national studies have confirmed that analgesic measures are infrequently implemented in neonatal ICUs.^(^^4,8,9^^)^

Pharmacological strategies consider the use of drugs to treat and relieve pain.^(^^10^^)^ Nonpharmacological strategies favor other modalities of care, especially during the modulation stage of the painful experience.^(^^7,10,11^^)^ Internationally established protocols subsidize the efficacy and safety of using these strategies among neonates.^(^^12,13^^)^

Given that the management of neonatal pain remains a challenge for healthcare practitioners, the present study aimed to describe and quantify the pharmacological and nonpharmacological strategies used to relieve pain and promote neonate comfort during their hospitalization in neonatal ICUs.

## METHODS

This longitudinal study collected data from the neonatal progressive care unit of a public maternity hospital located in Belo Horizonte (MG, Brazil) between February and June 2014. This philanthropic institution is located on the outskirts of the municipality, specializes in the healthcare of women and neonates, and exclusively serves the clients of the Brazilian Sistema Único de Saúde (SUS). At the time of this study, the maternity ward had 72 obstetric beds and averaged 9,300 deliveries per year. The mean number of hospitalizations in the neonatal ICU was 2,200 hospitalizations/year, with a mean length of stay of 12 days.

Infants born at the maternity hospital, admitted to the ICU or semi-ICU during their first 3 hours of life, and whose guardians/parents signed an informed consent document were included in this study. Neonates transferred to other institutions after their birth and those with major congenital malformations were excluded.

To collect the data for this study, a specific form was developed regarding information to characterize the neonates (date and time of birth, type of delivery, Apgar score at 1 and 5 minutes of life, sex, weight, gestational age at birth, and classification according to weight and gestational age and head circumference), their main clinical diagnoses (cause of hospitalization), the painful procedures performed, and the use and type of analgesia.

The present study considered venous and arterial puncture, capillary puncture, tracheal intubation, pulmonary mechanical ventilation, the introduction of drains, the aspiration of a tracheal cannula, a gastric probe, and the removal of adhesive tapes as painful procedures. The procedures considered as stressors were ventilation via positive pressure in the airways, respiratory physiotherapy, handling, hygiene, and weighing. The nonpharmacological pain-management strategies were the application of oral sweetened solutions (25% glucose), breastfeeding, skin-to-skin contact, facilitated containment, positioning, and brightness and noise control.

The applications of dipyrone, paracetamol, fentanyl, and morphine were considered as pharmacological interventions. Information regarding the use of sedatives (e.g., midazolam and chloral hydrate) were considered. Although these are not analgesic drugs, they are often used in isolation (which reflects a lack of knowledge regarding the difference between sedation and analgesia) or combined with analgesic drugs. The professionals of the unit established the indications for the use of pharmacological and nonpharmacological pain-management measures via evaluation or the empirical judgment of the clinical context without the interference of the researchers.

The nursing team staff who were responsible for directly assisting the neonates completed the data-collection forms daily. Every neonate in the study was followed up from admission to the neonatal ICU to discharge. As a preliminary step, training was offered to the nurses working in the participating neonatal ICUs to inform them of the objectives and procedures of the study as well as with regard to how to complete the data-collection instrument. In addition, a pilot study was conducted for 30 days to validate the instrument.

The data were entered twice into Microsoft Excel for Windows 2007 and analyzed using Minitab 15.1. To characterize the sample, a descriptive analysis was performed. The qualitative variables were presented as relative percentages and absolute (n) frequencies, considering each class of variable. Quantitative variables were represented as means, minimums, and maximums.

The study respected the recommendations of privacy and confidentiality set forth by Resolution Nº 466 of October 12, 2012 of the National Council for Scientific Research with Human Beings and was approved by the Ethics and Research Committee of the *Universidade Federal de Minas Gerais* and the field institution of this study.

## RESULTS

A total of 110 neonates were hospitalized during the study period, of whom 50 met the inclusion criteria. Of the neonates excluded, 10 were born at another service or at home, 15 were admitted to the neonatal ICU after 3 hours of life, 14 parents or guardians refused to sign the informed consent document, and the information of 21 neonates was incomplete on the data-collection instrument on admission. The demographic data of the neonates included in this study are shown in table 1.

**Table 1:** Demographic data of the neonates admitted to the neonatal intensive care unit

Demographic characteristics	
Gestational age (weeks)	30.76 (28 - 32)
Birth weight (g)	1,783 (765 - 2,105)
Sex (female/male)	18/32
Apgar score, 1 minute	6 (0 - 9)
Apgar score, 5 minutes	8 (4 - 10)
Type of delivery (vaginal/cesarean section)	19/31
Length of hospital stay (days)	21.56 (18/131)

Results presented as the mean (minimum/maximum), %, or median (minimum/maximum).

The clinical diagnoses prevalent at admission were prematurity (33.7%), respiratory distress (20.7%), neonatal respiratory distress syndrome (15.5%), and presumed early onset sepsis (10.3%). Other records (19.8%) included very low birth weight, neonatal anoxia, and transient tachypnoea.

During hospitalization, the neonates underwent 9,948 painful and stressful procedures (mean = 11.25 ± 6.3 procedures per day per neonate).

A total of 11,722 interventions were performed to treat and manage pain throughout the hospitalization of the neonates; of these cases. 11,495 (98.1%) were nonpharmacological, and 227 (1.9%) were pharmacological. Each neonate underwent an average of 235 pain-management and treatment interventions during hospitalization, 13 nonpharmacological interventions per day, and a pharmacological intervention every 2 days per neonate. Tables 2 and 3 present the nonpharmacological and pharmacological methods employed during the observation period.

**Table 2:** Nonpharmacological methods of pain management and treatment among neonates hospitalized in the neonatal intensive care unit

Nonpharmacological methods	n (%)
Positioning	2,962 (25.7)
Brightness reduction	2,341 (20.3)
Noise reduction	2,163 (18.8)
Minimal handling	1,439 (12.5)
Containment	1,317 (11.4)
Breastfeeding	395 (3.4)
Breast milk supply	358 (3.1)
Sweetened solution (25% glucose)	193 (1.7)
Nonnutritive sucking	171 (1.5)
Skin-to-skin contact	156 (1.4)
Total	11,495 (100.0)

**Table 3:** Pharmacological methods of pain management and treatment among neonates hospitalized in the neonatal intensive care unit

Pharmacological methods	n (%)
Analgesic drugs	
Intermittent fentanyl	72 (31.7)
Continuous fentanyl	60 (24.4)
Intermittent morphine	7 (3.1)
Continuous morphine	3 (1.3)
Intermittent dipyrone	8 (3.5)
Sedatives	
Intermittent midazolam	46 (20.3)
Continuous midazolam	26 (11.5)
Total	227 (100.0)

## DISCUSSION

This study described and quantified the pharmacological and nonpharmacological strategies used to relieve the pain of neonates during their hospitalization in neonatal ICUs. A total of 9,948 painful or stressful procedures were performed during the hospitalization of the 50 neonates included in this study. Each neonate was submitted to an average of 11.25 (±6.3) procedures per day. In routine care, there are great difficulties in recognizing pain and incorporating pain-management practices.^(^^4,9^^)^

Regarding pain management, the data from this study show that in most cases the care team used nonpharmacological pain-management strategies as the therapeutic option (98.1%). However, the alignment between the pain stimulus and the intervention was not the objective of the present study. The most frequently used nonpharmacological measures included nesting positioning (25.8%), environment control through the reduction of brightness (20.4%) and noise (18.8%), minimal handling (12.5%), and containment (11.4%). These strategies are inexpensive, easily assimilated and implemented by the multidisciplinary team, and have low or no risk of complication. Although they do not constitute specific care for the management of neonatal pain, these measures favor neuropsychomotor organization and act during the stage of pain modulation, thereby inhibiting the release of the neurotransmitters responsible for exacerbating the initial pain stimulus.^(^^7^^)^

Special attention should be paid to these aspects of care during the moments prior to performing painful procedures. During these procedures, the lights are commonly turned on and the noise tends to intensify while the neonates are handled and removed from a condition of comfort and organization, which accentuates the painful stimulation.^(^^6^^)^

Nonpharmacological measures can be adopted in isolation as one approach in cases of mild pain or as adjuvant strategies in the case of moderate-to-severe pain. Studies have shown that the combination of more than one nonpharmacological measure can present a synergistic protective effect, as in the case of nonnutritive sucking and oral glucose solution.^(^^7^^)^

However, the practices proven to effectively relieve procedural pain in neonates were rarely used. For example, breastfeeding reduces the pain of procedures such as venous and capillary punctures for blood collection and immunizations. Many systematic reviews support the effectiveness and safety of breastfeeding as an analgesic measure. In addition, the World Health Organization recognizes and recommends the use of this practice during immunization.^(^^14,15^^)^ To be effective, breastfeeding must be started approximately 5 minutes before the painful procedure; in addition, the neonate must suck effectively before, during, and after the end of the painful procedure so that effective analgesic effects are obtained.

A recently published systematic review synthesized the evidence regarding the effectiveness of skin-to-skin contact as an analgesic method for procedural pain among neonates. The results indicated that skin-to-skin contact is an effective and safe strategy for reducing the pain associated with isolated procedures. Ideally, the neonate should be positioned to receive skin-to-skin contact approximately 10 to 15 minutes before the procedure and should remain in this position until its completion.^(^^16-18^^)^

With regard to sweetened solutions, evidence supports the administration of glucose and sucrose as analgesic measures. Small volumes of glucose or sucrose administered on the anterior portion of the tongue of neonates approximately 2 minutes before a procedure guarantee the reduction of pain scores.^(^^17,19^^)^ Nonnutritive sucking promotes comfort and pain relief among preterm and term neonates and can be used alone or combined with sweetened solutions.^(^^17,20^^)^

According to the literature, certain difficulties restrict the use of these strategies in neonatal ICUs, including the following: the lack of knowledge among parents and professionals regarding the effectiveness and benefits of these strategies; professionals' belief that infants will associate painful events with breastfeeding or skin-to-skin contact; the orientation given to parents regarding the use of those resources; and the need to adjust technical procedures to use pain-relief strategies (e.g., the position to be adopted by professionals to immunize and perform punctures in breastfed neonates).^(^^12,13^^)^ The main barrier related to the use of glucose and nonnutritive sucking in this study, however, was the restriction related to the use of nonnutritive sucking (i.e., pacifier use) in hospitals certified by the *Hospital Amigo da Criança*.^(^^12^^)^

To overcome these barriers, knowledge socialization strategies are necessary to improve pain-management practices in neonatal ICUs. Traditionally, protocols and recommendations are published by governmental and nongovernmental organizations as well as societal and class entities. In addition, institutions adapt such protocols and recommendations based on the populations served and the resources available.

In addition, certain knowledge-based strategies focusing on the use of nonpharmacological measures for neonatal pain relief are available on virtual platforms such as YouTube. For example, the video *Seja Doce com os Bebês* (Be Sweet with Babies; https://youtu.be/ZGLSNdYtppo), shows the effects of breastfeeding, skin-to-skin contact, and sweetened solutions on the relief of procedural pain. Studies have indicated that parents and nurses consider this video to be useful as well as easy to understand and apply to real-life scenarios.^(^^21^^)^ Another video, The Power of a Parent's Touch (https://youtu.be/3nqN9c3FWn8), which features a subtitled version in Portuguese, is considered as useful by parents and health professionals with regard to changing practices.^(^^22^^)^

These knowledge-based tools can be implemented as family-oriented actions to provide knowledge and foster parent involvement in aspects of childcare, including pain management. In addition, they can be adopted as resources for the continuing education of health professionals involved in neonatal care.

Finally, the use of drugs to manage the pain of neonates included in this study was limited (1.9%) given the number of interventions received by the analyzed population. These data indicate difficulties in therapeutic indications that might be related to the absence of evaluation and treatment protocols, a lack of knowledge among the teams, or the myths that permeate clinical practice, which (despite recent advances) continue to encourage undertreatment.

The most effective analgesic therapies for moderate-to-severe pain for all age groups, and neonates in particular, attenuate the physiological responses related to stress.^(^^23,24^^)^ The intensity of the pain stimulus and the choice of the therapy to be used must be fine-tuned so that the benefits of the treatment outweigh the risks. The main discussion point regarding neonatology concerns the neurotoxicity of drugs, which is difficult to assess in humans but has proven results in animal models.^(^^19^^)^ Importantly, this argument does not support the absence of treatment because exposure to pain during the neonatal period can alter the brain's final architecture, with severe physiological, cognitive, and behavioral repercussions expressed throughout development.^(^^5^^)^

Midazolam is a benzodiazepine that has direct action on motor activity and agitation. However, it has no analgesic effect and should not be used in isolation to treat pain. The continuous infusion of midazolam in premature neonates can cause serious adverse effects such as death, leukomalacia, and peri-intraventricular hemorrhage. No studies have demonstrated contraindications to its intermittent use.^(^^23,24^^)^

The present study did not investigate the alignment between pain intervention and the indicated treatment; importantly, however, conceptual misconceptions are still observed in clinical practice because benzodiazepines and sedatives are prescribed and administered in isolation to treat pain. Thus, it is important to apply evaluation instruments and exclude the possibility of pain when these types of medicine are indicated.

The use of medication to manage the pain of neonates is challenging for the multidisciplinary team. Renal and hepatic immaturity as well as the risk of respiratory depression because of the use of opioids limits the use of drugs in clinical practice.^(^^24^^)^ For these difficulties not to lead to a lack of treatment when indicated, the multidisciplinary team should discuss and validate clinical protocols based on scientific evidence, and the therapeutic interventions appropriate to each procedure should be defined.

Regarding the study limitations, we highlight the absence of local protocols to evaluate and treat neonatal pain at the institution where the data were collected; the nonalignment between the pharmacological and nonpharmacological strategies; the painful intervention; and the incomplete filling of the data-collection instrument by the assistant team, which was minimized by the monitoring and data recovery performed by the researchers involved during this phase.

## CONCLUSION

During hospitalization, neonates primarily receive nonpharmacological pain-relief strategies. However, indications of pharmacological measures of pain relief remain a challenge regarding the care of hospitalized neonates. Thus, effective evaluation protocols must be implemented to adequately manage pain.

With regard to the nursing team, it is essential to increase their knowledge and the feasibility of the evaluations and implementations of nonpharmacological and pharmacological strategies for neonatal pain relief.
